# Halophilic Bacteria of Lunsu Produce an Array of Industrially Important Enzymes with Salt Tolerant Activity

**DOI:** 10.1155/2016/9237418

**Published:** 2016-01-18

**Authors:** Sonika Gupta, Parul Sharma, Kamal Dev, Anuradha Sourirajan

**Affiliations:** Faculty of Applied Sciences and Biotechnology, Shoolini University of Biotechnology and Management Sciences, Solan, Himachal Pradesh 173212, India

## Abstract

The halophilic bacterial isolates SS1, SS2, SS3, SS5, and SS8 were characterized for production of industrially important enzymes like amylase, protease, lipase, and glutaminase. Halophilic bacterial isolates SS1 and SS3 exhibited salt dependent extracellular amylase and protease activities. Both the halophilic isolates SS1 and SS3 exhibited maximum amylase and protease activities in the presence of 1.5 and 1.0 M NaCl, respectively, with the optimum pH 8 and temperature 40°C. SS2 showed maximum extracellular protease and lipase activities in the presence of 0.75 M NaCl, at optimum pH of 7, and temperature 37°C. The glutaminase activity of SS3 increased with increase in concentration of NaCl up to 2.5 M. The optimum pH and temperature for L-glutaminase activity of SS3 was 8 and 40°C, respectively. The combined hydrolytic activities of these halophilic bacterial isolates can be used for bioconversion of organic materials to useful products.

## 1. Introduction

Hydrolases constitute a class of enzymes widely distributed in nature from bacteria to higher eukaryotes. The growing demand for enzymes stable under high salt concentrations has focused attention on halophiles. Halophiles receive special interest because they can grow in a wide range of salt concentrations. Halophilic microorganisms are a potential source of extremozymes called halozymes like proteases, amylases, nucleases, lipases, cellulases, xylanases, catalases, and esterases, which are capable of functioning under high concentrations of salt, wide range of pH values, and temperatures at which other proteins will usually precipitate or denature. The halozymes can be exploited wherever enzymatic transformations are required to function in the presence of organic solvents and extremes in temperature and salt content. Halozymes have identical enzymatic features like their nonhalophilic counterpart but exhibit marked differences in their structural features which make them functional at harsh conditions [1]. These include high proportion of acidic amino-acids located predominantly at the protein surface and requirement of high salt concentration for optimum biological functions. The halophilic enzymes are also stable in the presence of high salt concentrations due to conglomeration of slightly hydrophobic groups and hydration of the protein surface due to carboxylic groups present in glutamate and aspartate. These halozymes have been commercialized in various industries including food, baking, feed, chemical and pharmaceutical, paper and pulp, detergent, leather industries, fish sauce and soy sauce preparations, saline waste water, and oilfield waste treatment [2, 3]. Various halophiles such as* Salicola marasensis* sp. IC10,* Pseudoalteromonas* spp.,* Haloarcula sp*.,* Haloferax mediterranei*,* Natrialba magadii*,* Salimicrobium halophilum* LY20,* Salinivibrio sp*.,* Nesterenkonia* sp. strain F,* Chromohalobacter sp*. TVSP 101, and* Halomonas meridiana* have been explored for halozymes such as amylase, lipase, glutaminase, and protease [4–8]. Certain enzymes from halophiles display polyextremophilic features like haloalkaliphilicity, thus showing activity in high saline and high alkaline conditions [9]. For example, enzymes isolated from* Pseudoalteromonas* spp. CP76 show its optimum activity at high salt concentration (7.5% NaCl) and pH 8.5 [2]. In addition, some archaeal enzymes are of great interest, such as the amylase of* Haloarcula* sp., function optimally at 4.3 M salt and stable in toluene, benzene, and chloroform [10].

As salt has the effect of reducing water activity, halophilic enzymes are thought to be important biocatalysts in low-water-activity environments, such as in aqueous/organic and in nonaqueous media [11]. An alkaline protease tolerant to xylene, ethanol, acetone, butanol, benzene, and chloroform has been reported from salt tolerant* Streptomyces clavuligerus* strain Mit-1 [12].

In this study we describe the screening and characterization of the extracellular hydrolytic enzymes produced by halophilic microorganisms isolated from the soil sediment of Lunsu, a salt water body of Himachal Pradesh, India. To the author's knowledge, this is the first study on the evaluation of extracellular hydrolytic activities of microorganisms isolated from Lunsu.

## 2. Materials and Methods

The halophilic bacterial strains used in this study were isolated from soil sediment of Lunsu water body, Himachal Pradesh, India [13]. The bacterial isolates were identified by 16S rDNA sequencing using 27 F and 1492 R primers.

Five halophilic bacterial strains* Halobacillus trueperi* SS1 (KM260166),* Halobacillus trueperi* SS3 (KF751761),* Shewanella algae* SS2 (KF751760),* Halomonas venusta* SS5 (KF751762), and* Marinomonas sp*. SS8 (KF751763) were used for screening and characterization of extracellular halozymes.

### 2.1. Characterization of Enzymatic Features of Halophilic Bacteria

#### 2.1.1. Qualitative Enzyme Assays

The halophilic bacterial isolates SS1, SS2, SS3, SS5, and SS8 were screened for amylase, protease, and lipase activity on LB agar medium containing 1 M NaCl. Medium was supplemented with 1% starch, 1% skim milk, and 1% Tween-80 for respective amylase, protease and lipase activities. All the halobacterial isolates were cultured in LB with 1 M NaCl by incubating at 37°C for 24 h with shaking at 200 rpm. The absorbance of the culture was measured at 600 nm and cell density was normalized to *A*_600_ = 0.1. Equal number of cells of each strain was spotted and the plates were incubated at 37°C for 48 hours.

These halophilic isolates were also used for the detection of L-glutaminase production by dye based procedure [14]. All the bacterial cultures were inoculated in medium supplemented with 1% L-glutamine and phenol red pH indicator. The culture tubes were incubated at 37°C for 48 h.

For visualizing amylase activity, the starch agar plates having bacterial growth were flooded with Gram's iodine solution. The appearance of clear zone around the bacterial colonies indicated the utilization of starch by the halophilic bacteria and thus indicative of the presence of amylase activity. The plates were assessed for protease activity by monitoring the zone of clearance around the bacterial growth. The zone formation around the colony indicates the hydrolysis of casein present in skim milk due to protease production. Bacteria producing lipase exhibited precipitation of Tween-80 around the region of bacterial growth and zone of precipitation around colonies on Tween-80 agar plates confirmed that the isolates produced lipase enzyme. For each assay, the zone of clearance was measured from three independent experiments and the average values (in cm) are reported with standard deviation. The glutaminase production was assessed by the change in color of medium from yellow to pink.

### 2.2. Quantitative Enzyme Assays

#### 2.2.1. Preparation of Extracellular Crude Enzyme

The halophilic isolates producing the respective enzymes were inoculated in LB broth containing 1 M NaCl and cultured at 37°C for 48 hours. After incubation period, all the cultures were centrifuged at 8000 rpm at 4°C for 15 min. The cell-free spent medium of the cultures was used to determine the extracellular enzyme activity. Total proteins in cell-free spent medium were estimated by Bradford method [15].

#### 2.2.2. Amylase Activity

Amylase activity was quantified by Dinitrosalicylic acid (DNS) method using starch as a substrate [16]. 20 *μ*g of total protein (cell-free spent medium as extracellular enzyme source) was added to 0.2 mL of 1% starch solution. The reaction mixture was incubated at 37°C for 30 min and the enzyme reaction was stopped by the addition of 2.0 mL DNS reagent. Further, reaction mixtures were boiled in a water bath for 10 min, cooled at room temperature, and the absorbance was measured at 540 nm. One unit of amylase was defined as the amount of enzyme liberating 1 *μ*g of maltose per minute under the assay conditions.

#### 2.2.3. Protease Activity

Protease activity was measured as described by Kuberan et al. [17]. The reaction mixture containing 1 mL of 1% casein (insoluble) in reaction buffer (50 mM Tris Cl, pH 8.0, and 100 mM NaCl) was mixed with 20 *μ*g of protein as crude cell-free enzyme source and incubated at 37°C for 30 minutes. The reaction was stopped by adding 3 mL of 5% trichloroacetic acid (TCA), followed by centrifugation at 8,000 rpm for 5 min. The supernatant was used for quantification by Lowry's method using tyrosine as a standard by measuring absorbance at 660 nm [18]. One unit of protease activity was defined as the amount of enzyme that liberates 1 *μ*g of tyrosine per mL of the reaction mixture per minute under the assay conditions.

#### 2.2.4. Glutaminase Activity

The glutaminase enzyme activity was measured by estimating the amount of ammonia liberated from glutamine [19]. 20 *μ*g of protein as crude cell-free enzyme source was mixed with 0.5 mL of 0.04 M L-glutamine, 0.5 mL of 0.1 M phosphate buffer (pH 7.0), and 0.5 mL of distilled water. The reaction mixture was incubated at 37°C for 30 min and stopped by adding 0.5 mL of 1.5 M TCA. 0.5 mL of above mixture was taken and added to 3.7 mL of distilled water followed by addition of 0.2 mL Nessler's reagent. The absorbance was recorded at 450 nm and the amount of ammonia liberated was calculated from the standard graph of ammonium sulphate. One unit of glutaminase was defined as amount of enzyme that liberates one micromole of ammonia under assay conditions.

#### 2.2.5. Lipase Activity

Lipase assay mixture consisted of 0.75 mM para nitrophenyl palmitate (pNPP) and 20 *μ*g of crude cell-free enzyme in 0.1 M potassium phosphate buffer of pH 7.0 in a 3 mL reaction volume. Reaction mixture was incubated at 37°C for 30 min and stopped by adding 1 mL chilled mixture of acetone : ethanol (1 : 1). The absorbance of p-nitrophenol released was measured at 410 nm [20]. The unknown concentration of p-nitrophenol released was determined from the standard graph of p-nitrophenol. One unit of lipase was defined as the amount of enzyme that releases one micromole of p-nitrophenol per minute under assay conditions. Enzyme blank was prepared in all the assays without adding the respective enzyme.

### 2.3. Determination of the Optimal NaCl Concentration, pH, and Temperature for Enzymes' Activities

Under standard assay conditions described above, the optimal NaCl concentrations, pH, and temperature for each of the enzyme's (amylase, protease, glutaminase, and lipase) activity were determined. To study the effect of NaCl on enzyme activities, enzyme assays were performed at varying NaCl concentrations (0, 0.5, 1, 1.5, 2, and 2.5 M NaCl). Optimal temperature for enzyme activity was determined by incubating the reaction mixture at different temperatures within the range of 20–60°C. Effect of pH on the different enzyme activity was studied in reaction buffer of varying pH (5–11). Reaction buffers used include citrate-phosphate buffer (pH 5 and 6), phosphate buffer (pH 7), Tris-HCl buffer (pH 8 and 9), and glycine-NaOH buffer (pH 10 and 11).

All the enzyme assays were repeated at least three times, and the results reported here represent the average from three independent experiments with standard deviation.

## 3. Results

### 3.1. Halophilic Bacterial Isolates SS1 and SS3 Have Salt Dependent Halophilic Amylase Activity

**Figure 1:**
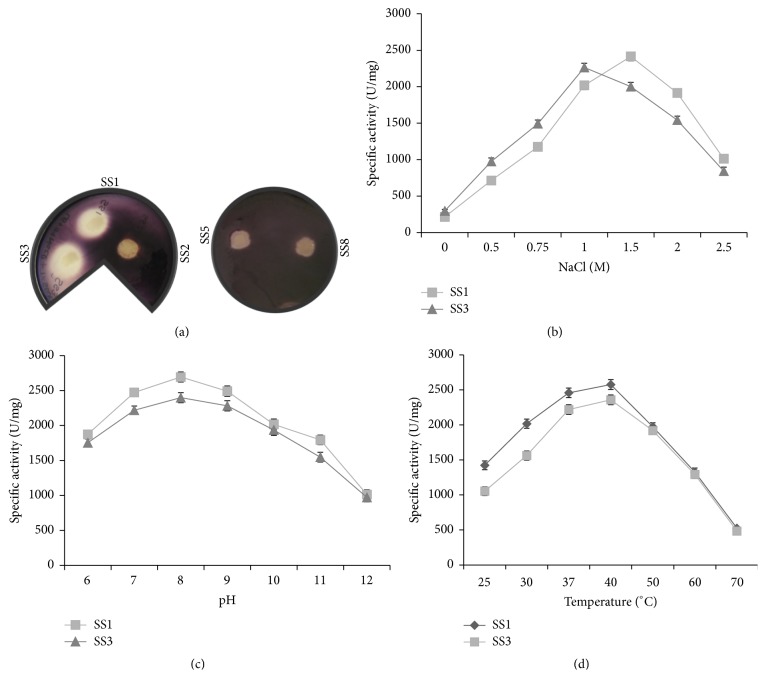
Screening and characterization of halophilic bacterial isolates for amylase activity. (a) Equal number of cells of indicated halophilic bacterial isolates (SS1, SS2, SS3, and SS5 and SS8) were spotted on Luria Bertani (LB) agar medium supplemented with 1% starch and 1 M NaCl. The plates were incubated at 30°C for 24 h and flooded with Gram's iodine to visualize zone of clearance due to starch digestion by amylase. Effect of NaCl, pH, and temperature on amylase activity of halophilic isolates: the specific amylase activity (U/mg) of the bacterial isolates* Halobacillus trueperi* SS1 and SS3 was plotted against the different concentrations of sodium chloride (NaCl) (b), varying pH (c), and temperature (d) as indicated. In each panel, the data represent an average of three independent experiments with standard deviation.

To check the ability of halophilic bacterial isolates for amylase production, starch agar plates having bacterial growth were flooded with Gram's iodine as described above. After flooding with Gram's iodine, a clear zone was observed around the growth of halophilic isolates SS1 and SS3, which indicated the presence of amylase activity. The average diameter of zone of clearance was 1.8 ± 0.5 cm for SS1 and 2.0 ± 0.4 cm for SS3, respectively (Figure 1(a)). In contrast, no zone of clearance was observed for SS2, SS5, and SS8 isolates.* E*.* coli* strain DH5*α* neither exhibited growth nor any amylase activity.

### 3.2. Effect of Salt (NaCl), pH, and Temperature on the Amylase Activity of Halophilic Bacterial Isolates

Quantitative amylase assay was carried out by DNS method using starch as a substrate and cell-free spent medium as enzyme source as described. Enzyme assay was performed with the amylase producing halophilic bacterial isolates, SS1 and SS3. To study the effect of salt on amylase activity, quantitative amylase assay was performed in reaction buffer containing increasing concentrations of NaCl (0, 0.5, 0.75, 1.0, 1.5, 2.0, and 2.5 M). Halophilic bacterial isolates SS1 and SS3 showed extracellular amylase activity in the presence of 0.5–2 M NaCl, with maximum enzyme activity of 2416 ± 57 U/mg at 1.5 M and 2264 ± 78 U/mg at 1 M NaCl, respectively (Figure 1(b)). Interestingly, no amylase activity was observed in the absence of NaCl (0 M) for both SS1 and SS3, indicating halophilic nature of their amylase activity (Table 1).

The extracellular amylolytic activity of SS1 and SS3 was assayed at different temperatures ranging from 25 to 70°C and pH 6–12 in the presence of their optimum NaCl concentration (1.5 M for SS1 and 1 M for SS3). Both the halophilic isolates (SS1 and SS3) showed amylase activity in a pH range of 6–12 and temperature range of 25–60°C (Figures 1(c) and 1(d)). The optimum pH and temperature for amylase activity of SS1 and SS3 were found to be 8 and 40°C, respectively (Table 1). Surprisingly, 60–63% amylase activity was retained at pH 12 and 50–52% amylase activity was observed at 60°C (Figures 1(c) and 1(d)).

### 3.3. The Halophilic Bacterial Isolates SS1, SS2, and SS3 Have Protease Activity

**Figure 2:**
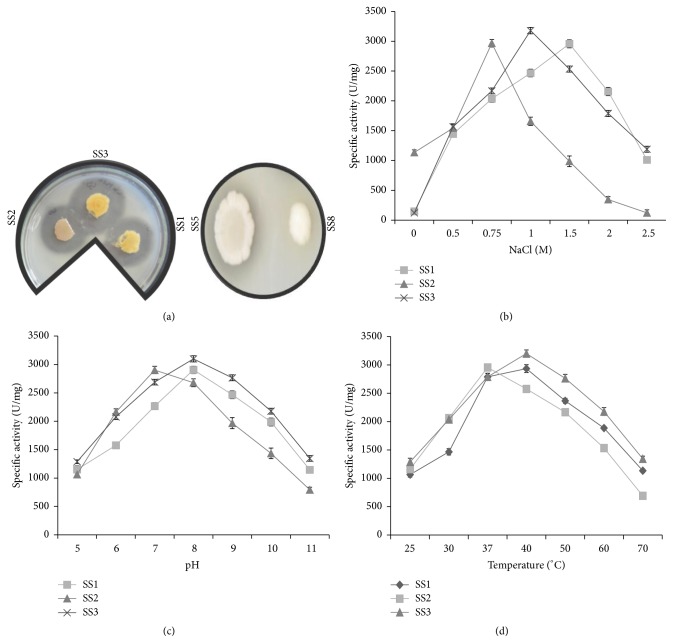
Screening and characterization of halophilic bacterial isolates for protease activity. (a) Equal number of cells of all halophilic isolates (SS1, SS2, SS3, SS5, and SS8) were spotted on LB agar medium supplemented with 1% skim milk and 1 M NaCl. The plates were incubated at 30°C for 24 hours to visualize the zone of clearance due to protease activity. Effect of NaCl, temperature, and pH on protease activity of halophilic isolates: the specific protease activity (U/mg) of the bacterial isolates* Halobacillus trueperi* SS1,* Shewanella algae* SS2, and* Halobacillus trueperi* SS3 was plotted against the different concentrations of sodium chloride (NaCl) (b), varying pH (c), and temperature (d) as indicated. In each panel, the data represent an average of three independent experiments with standard deviation.

Most halophilic bacteria are known to secrete proteases into the external environment that can have unique applications in biotechnology. The halophilic isolates were therefore screened for protease production based on the zone of clearance surrounding the growth on skimmed milk agar plates (LB agar medium supplemented with 1 M NaCl and 1% skim milk) at 37°C as described above. The zone of clearance appears due to the digestion of casein present in skimmed milk by the action of extracellular protease. Halophilic bacterial isolates SS1, SS2, and SS3 were found protease positive, while the isolates SS5, SS8, and* E*.* coli* strain DH5*α* were found to be protease negative (Figure 2(a)). The diameter of zone of clearance of SS1, SS2, and SS3 was 1.9 ± 0.3, 2.5 ± 0.2, and 2.1 ± 0.4 cm, respectively. Therefore, halophilic bacterial isolates SS1, SS2, and SS3 were used to measure their protease activity.

### 3.4. Effect of NaCl, pH, and Temperature on the Protease Activity of Halophilic Bacterial Isolates

To assess the halo tolerant nature of protease enzyme, quantitative protease assay was performed (as described) in a reaction buffer containing increasing concentrations of NaCl (0, 0.5, 0.75, 1.0, 1.5, 2.0, and 2.5 M). The protease activity of isolate SS1 and SS3 increased with increasing concentration of salt in the reaction, with maximum activity in the presence of 1.5 and 1 M NaCl, respectively (Figure 2(b); Table 1). The maximum protease activity of SS1 and SS3 was 2956 ± 52 and 3178 ± 89 U/mg, respectively (Figure 2(a)). No activity was observed in the absence of NaCl and almost 50% activity was retained at 2 M NaCl. These results indicate that the protease activity of SS1 and SS3 isolates is halophilic in nature. It was observed that bacterial isolate SS2 showed maximum protease activity of 2967 ± 49 U/mg in the presence of 0.75 M NaCl.

The effect of pH and temperature on caseinolytic activity of the enzyme was determined in presence of optimal NaCl concentration. To determine the optimum pH for protease activity, protease assay was carried out in a reaction buffer of varying pH (5–12). Protease activity of halophilic isolate SS1, SS2, and SS3 was observed between pH 6 and 11. The maximum protease activity was observed at pH 8.0 for SS1 and SS3 isolate, whereas it was pH 7.0 for SS2 (Figure 2(c); Table 1). These results indicate that the extracellular protease from halophilic isolate SS2 was neutrophilic in nature.

To determine the effect of temperature on protease activity, protease assay was performed as described above and incubated at different temperatures (25, 30, 37, 40, 50, 60, and 70°C). The maximum protease activity of SS1 and SS3 isolates was observed at 40°C, whereas SS2 isolate exhibited maximum protease activity at 37°C, with 30% activity retained at 60°C. These results were suggestive of a broad range of temperature for protease activity (Figure 2(d)).

### 3.5. The Halophilic Bacterial Isolate SS2 Exhibits Halotolerant Lipase Activity

**Figure 3:**
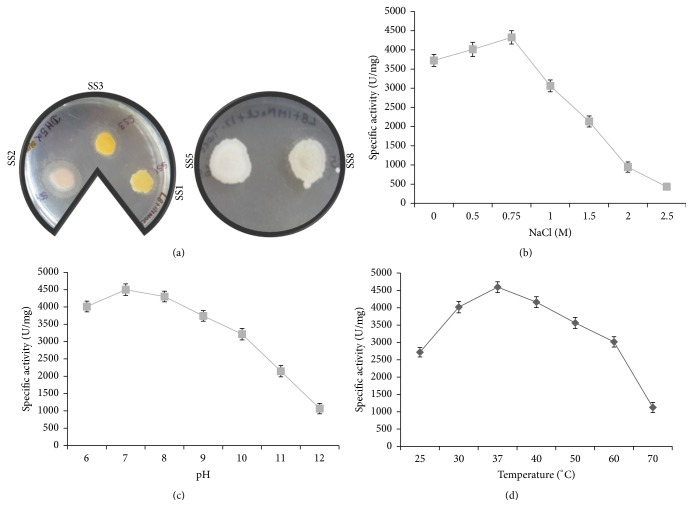
Screening and characterization of halophilic bacterial isolates for lipase production. (a) Equal number of cells of halophilic bacterial isolates (SS1, SS2, SS3 and SS4, and SS5 and SS8) was spotted on LB agar medium supplemented with 1% Tween-80 and 1 M NaCl. The plates were incubated at 30°C for 24 h to visualize lipase activity. Effect of NaCl, pH, and temperature on lipase activity of halophilic isolates: the specific lipase activity (U/mg) of the bacterial isolate* Shewanella algae* SS2 was plotted against the different concentrations of sodium chloride (NaCl) (b), varying pH (c), and temperature (d) as indicated. In each panel, the data represent an average of three independent experiments with standard deviation.

To detect the production of lipase enzymes, the halobacterial isolates (SS1, SS2, SS3, SS5, and SS8) were spotted on LB medium supplemented with 1 M NaCl and 1% Tween-80 as described. Only the halobacterial isolate SS2 produced zones of precipitation that indicates the presence of lipase activity (Figure 3(a)). On the other hand,* E*.* coli* strain DH5*α* failed to grow in the medium supplemented with NaCl.

### 3.6. Effect of NaCl, pH, and Temperature on the Lipase Activity of Halophilic Bacterial Isolate

To assess the halo tolerant nature of lipase activity of SS2 isolate, quantitative enzyme assay was carried out in a reaction buffer containing increasing concentrations of NaCl (0–2.5 M) as described. Enzyme activity was determined by using pNPP as substrate. The halophilic bacterial isolate SS2 showed maximum lipase activity of 4327 ± 92 U/mg in the presence of 0.75 M NaCl, and the lipase activity decreased with increase in NaCl concentration above 0.75 M NaCl (Figure 3(b)).

The effect of pH and temperature on lipase activity was determined in presence of optimal NaCl concentration. Maximum lipase activity of SS2 isolate was observed at pH 7.0 and temperature 37°C (Figures 3(b) and 3(c); Table 1). At pH 12, there was a drastic decrease in lipase activity and only 23–27% of lipase activity was retained (Figure 3(c)). The maximum lipase activity of isolate SS2 was observed at 37°C, with 65–72% activity retained at 60°C (Figure 3(d)). These results indicate that lipase enzyme is active over a wide range of temperature.

### 3.7. All the Halophilic Bacterial Isolates Except SS1 Exhibit Glutaminase Activity

**Figure 4:**
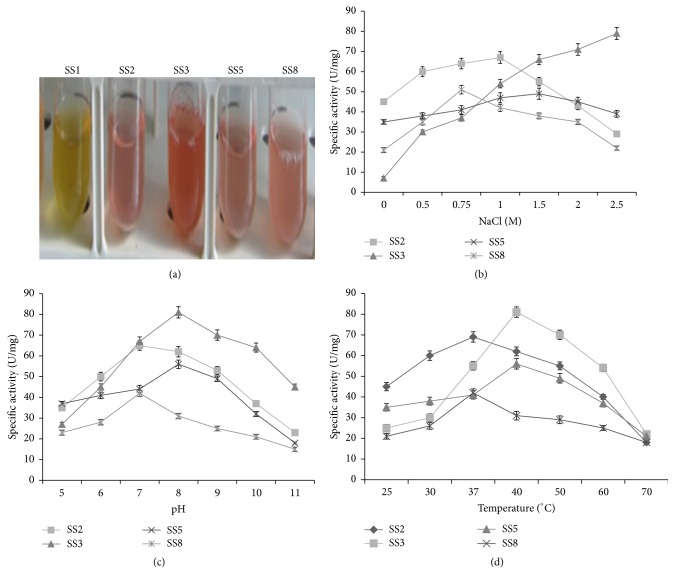
Screening and characterization of halophilic bacterial isolates for glutaminase production. (a) Equal number of cells of all halophilic bacterial isolates was inoculated in LB medium supplemented with 1% L-glutamine, phenol red, and 1 M NaCl the medium. The tubes were incubated at 30°C for 24 hours to visualize glutaminase activity. The change in colour of medium from yellow to pink indicates glutaminase activity. Effect of NaCl, pH, and temperature on glutaminase activity of halophilic isolates: the specific glutaminase activity (U/mg) of the bacterial isolates* Shewanella algae* SS2,* Halobacillus trueperi* SS3,* Halomonas venusta* SS5, and* Marinomonas* sp. SS8 was plotted against the different concentration of sodium chloride (NaCl) (b), varying pH (c), and temperature (d) as indicated. In each panel, the data represent an average of three independent experiments with standard deviation.

As glutaminase is an industrially important enzyme, therefore, the halophilic bacterial isolates were screened for glutaminase production as described above. All the halophilic bacterial isolates, except SS1 showed change in color of medium from yellow to pink (Figure 4(a)). These results indicate that these isolates are positive for glutaminase activity.

### 3.8. Effect of Salt, pH, and Temperature on the Glutaminase Activity of Halophilic Bacterial Isolates

The glutaminase activity of all halophilic isolates increased with increase in NaCl concentration up to 1 M NaCl (Figure 4(a)). The maximum specific glutaminase activity of bacterial isolates SS2, SS3, SS5, and SS8 at 1, 2.5, 1.5, and 0.75 M NaCl was 68 ± 3, 79 ± 4, 55 ± 3, and 41 ± 2 U/mg, respectively. Interestingly, glutaminase activity of SS3 increased with increase in NaCl concentration up to 2.5 M NaCl (Figure 4(b)). The optimum pH for glutaminase activity of bacterial isolate SS3 and SS5 was pH 8 (Figure 4(c)). Maximum glutaminase activity of SS2 and SS8 was observed at pH 7 (Figure 4(d)). The optimum temperature for glutaminase activity of SS3 and SS5 was 40°C, while it was 37°C for SS2 and SS8 (Table 1). More than 25% glutaminase activity of halophilic bacterial isolates was retained at pH 11 and temperature 70°C (Figure 4(d)).

## 4. Discussion

### 4.1. Halozymes Are Produced by Halophilic Bacterial Isolates of Lunsu

Halophiles are rich sources of enzymes such as protease, amylase, lipase, and xylanases. Collectively, these enzymes are referred to as halozymes, enzymes that exhibit salt tolerant or salt dependent catalytic activity. In the present study, the five halophilic bacterial isolates were found to produce one or more halozymes like amylase, protease, lipase, and glutaminase. In a hypersaline lake of Iran, Rohban et al. reported the ability of halophilic strains to produce different extracellular hydrolases (lipase, amylase, protease, xylanase, DNase, inulinase, pectinase, cellulase, and pullulanase) [21].* Virgibacillus* sp. strain JS5 isolated from the Arabian soil of west coast of Karnataka, India, exhibited potential to produce the extracellular enzymes such as amylase, protease, inulinase, and gelatinase [22].

Amylases are important component of several industries. Halophilic amylase offers an additional potential of being salt tolerant. In this study, SS1 and SS3 exhibited halophilic amylase activity, which required the presence of at least 0.5 M NaCl for enzyme activity. In quantitative assays, it was found that amylase activity of halophilic isolates SS1 and SS3 was highest in the presence of 1.5 and 1 M NaCl, respectively. This is an attractive property for saline waste water treatment containing starch residues in the presence of high salt. Moreover, no amylase activity was observed below 0.5 M NaCl. Optimum temperature and pH for amylase activity of SS1 and SS3 was 40°C and pH 8, respectively. Coronado et al. reported the optimum temperature of the *α*-amylase produced by* Halomonas meridiana* to be 37°C [23].* Halobacillus* sp. strain MA-2 was found to exhibit maximum amylase activity in the presence 1 M NaCl, and the optimum pH and temperature for enzyme activity was 7.5 and 50°C, respectively. A moderately halophilic strain LY9 with amylolytic activity was isolated from soil sample collected from Yuncheng, China. The enzyme showed optimal activity at 60°C, pH 8.0, and 2 M NaCl [24].

Halophilic/halotolerant protease enzymes are advantageous in industrial processes where the concentrated salt solution used will inhibit the nonhalophilic protease activity. Amongst an array of five halophilic bacterial isolates of Lunsu, halophilic bacterial isolates SS1, SS2, and SS3 exhibited extracellular proteolytic activity. Maximum protease activity of SS1 and SS3 was observed in the presence of 1–1.5 M NaCl, whereas SS2 showed maximum protease activity in presence of 0.75 M NaCl at pH 7. The presence of salt tolerant protease activity has been reported for* Halobacterium halobium*,* Natrialba magadii*, and* Natronococcus occultus* [25–27]. In another study, caseinolytic activity of the* Halobacillus karajensis* strain MA-2 was found to be optimal at temperature 50°C, pH 9.0, and 0.5 M NaCl [28]. In another study, optimum protease activity of a haloalkaliphilic bacteria S5 isolated from saline habitat of coastal Gujarat, India, was observed at 1.8 M NaCl and pH 9–9.5 [29]. Thus, comparison of the results from our study with the existing literature revealed that protease produced by halophilic bacterial isolates SS1 and SS3 exhibit the best halotolerance, being active up to 1.5 M NaCl.

Owing to their ability to function in the presence of organic solvents, halophilic lipases are advantageous as lipase mediated catalysis occurs at interface of aqueous and organic layers. Since lipases are advantageous for several applications, the halophilic bacterial isolates of Lunsu were explored for lipase production. SS2 isolate was found to possess extracellular lipase activity.

The optimum salt concentration, pH, and temperature for lipase activity of bacterial isolate SS2 were 0.75 M NaCl, pH 7.0, and temperature 37°C, respectively. Moderate halophiles of the genera* Bacillus* and* Staphylococcus* isolated from Maharlu salt lake in Iran were reported positive for lipase activity [30]. Extreme halotolerant lipase was also reported from* Haloarcula marismortui* [31, 32]. A halophilic strain* Chromohalobacter* sp. LY7-8 with high lipolytic activity was isolated from salt lake of Yuncheng, China [33]. The enzyme showed optimal activity at 60°C, pH 9.0, and 2.1 M NaCl. Similarly, halophilic lipase from the halotolerant* Staphylococcus warneri* PB233 showed optimum pH of 7.0 and temperature of 40°C for its activity. It was stable between pH 7.0 and 9.0 and temperature of 30–40°C. The purified lipase showed maximum activity in the presence of 2.5 M NaCl, indicating its halophilic nature [34]. Thus, the halophilic bacterial isolate SS2 can be exploited for production of halotolerant extracellular lipase.

Glutaminases, in general, exhibit activity in the presence of high concentration of salt. Halophiles can be a valuable source of glutaminase, owing to their production of halozymes. In the present study, halophilic bacterial isolates SS2, SS3, SS5, and SS8 exhibited the ability to secrete extracellular glutaminase with diverse properties. The glutaminase activity of halophilic bacterial isolates SS2, SS3, SS5, and SS8 was found to be salt tolerant, and the enzyme remained functional in the presence of 2.5 M NaCl. In addition, the haloenzyme glutaminase also showed activity in the presence of wide range of pH (6–11) and temperature (25–50°C).

Glutaminase from* Stenotrophomonas maltophilia* NYW-81 was found to be stable in the presence of 2.6 M NaCl at pH 8 [35]. About 50% of enzyme activity was retained at 70°C, for 10 min. L-Glutaminase obtained from* Aspergillus oryzae* showed optimum activity at temperature range of 37–45°C and lost its activity at 55°C [36]. The marine* Micrococcus luteus K3* was reported to have glutaminase activity at pH 8.0, temperature of 50°C, and 1.5–2 M NaCl [37]. Although glutaminase has been studied in fungi and other mesophilic bacteria, very few reports exist on glutaminase from halophiles. In this context, the halophilic bacterial isolates of Lunsu serve as a novel source of glutaminase from the halophilic domain.

## 5. Conclusion

The cost effective extracellular enzymes produced by halophilic isolates of Lunsu have great economical potential in industrial, agricultural, chemical, pharmaceutical, and biotechnological applications. The combined hydrolytic activity of some halophilic bacterial isolates can be used for bioconversion of organic materials to useful products in hypersaline or polluted environments. Owing to their ability to remain functional in extreme conditions such as high temperatures, wide range of pH, and high salt concentrations, the halozymes isolated in this study offer important biotechnological potential.

## Figures and Tables

**Table 1:** Biochemical characterization of halozymes. The optimum conditions for the respective enzyme activities are indicated.

Isolate	Amylase			Protease			Lipase			Glutaminase		
	NaCl (M)	pH	Temperature (°C)	NaCl (M)	pH	Temperature (°C)	NaCl (M)	pH	Temperature (°C)	NaCl (M)	pH	Temperature (°C)
SS1	1.5	8	40	1.5	8	40	−	−	−	−	−	−
SS2	−	−	−	0.75	7	37	0.75	7	37	1	7	37
SS3	1	8	40	1	8	40	−	−	−	2.5	8	40
SS5	−	−	−	−	−	−	−	−	−	1.5	8	37
SS8	−	−	−	−	−	−	−	−	−	0.75	7	37

Negative (−) sign indicates no detectable enzyme activity was observed.

## References

[1] Kamekura M., Enache M. (2010). Hydrolytic enzymes of halophilic microorganisms and their economic values. *The Journal of Biochemistry*.

[2] Sánchez-Porro C. S., Martín S., Mellado E., Ventosa A. (2003). Diversity of moderately halophilic bacteria producing extracellular hydrolytic enzymes. *Journal of Applied Microbiology*.

[3] Oren A. (2010). Industrial and environmental applications of halophilic microorganisms. *Environmental Technology*.

[4] Amoozegar M. A., Salehghamari E., Khajeh K., Kabiri M., Naddaf S. (2008). Production of an extracellular thermohalophilic lipase from a moderately halophilic bacterium, *Salinivibrio* sp. strain SA-2. *Journal of Basic Microbiology*.

[5] Ruiz D. M., De Castro R. E. (2007). Effect of organic solvents on the activity and stability of an extracellular protease secreted by the haloalkaliphilic archaeon *Natrialba magadii*. *Journal of Industrial Microbiology and Biotechnology*.

[6] De Lourdes Moreno M., García M. T., Ventosa A., Mellado E. (2009). Characterization of *Salicola* sp. IC10, a lipase- and protease-producing extreme halophile. *FEMS Microbiology Ecology*.

[7] Prakash B., Vidyasagar M., Madhukumar M. S., Muralikrishna G., Sreeramulu K. (2009). Production, purification, and characterization of two extremely halotolerant, thermostable, and alkali-stable *α*-amylases from *Chromohalobacter* sp. TVSP 101. *Process Biochemistry*.

[8] Li X., Yu H.-Y. (2012). Purification and characterization of novel organic-solvent-tolerant *β*-amylase and serine protease from a newly isolated *Salimicrobium halophilum* strain LY20. *FEMS Microbiology Letters*.

[9] Gupta A., Roy I., Patel R. K., Singh S. P., Khare S. K., Gupta M. N. (2005). One-step purification and characterization of an alkaline protease from haloalkaliphilic *Bacillus* sp.. *Journal of Chromatography A*.

[10] Fukushima T., Mizuki T., Echigo A., Inoue A., Usami R. (2005). Organic solvent tolerance of halophilic *α*-amylase from a haloarchaeon, *Haloarcula* sp. strain S-1. *Extremophiles*.

[11] Marhuenda-Egea F. C., Bonete M. J. (2002). Extreme halophilic enzymes in organic solvents. *Current Opinion in Biotechnology*.

[12] Thumar J. T., Singh S. P. (2009). Organic solvent tolerance of an alkaline protease from salt-tolerant alkaliphilic *Streptomyces clavuligerus* strain Mit-1. *The Journal of Industrial Microbiology and Biotechnology*.

[13] Gupta S., Sharma P., Dev K., Srivastava M., Sourirajan A. (2015). A diverse group of halophilic bacteria exist in Lunsu, a natural salt water body of Himachal Pradesh, India. *SpringerPlus*.

[14] Gulati R., Saxena R. K., Gupta R. (1997). A rapid plate assay for screening L-asparaginase producing micro-organisms. *Letters in Applied Microbiology*.

[15] Bradford M. M. (1976). A rapid and sensitive method for the quantitation of microgram quantities of protein utilizing the principle of protein-dye binding. *Analytical Biochemistry*.

[16] Miller G. L. (1959). Use of dinitrosalicylic acid reagent for determination of reducing sugar. *Analytical Chemistry*.

[17] Kuberan T., Sangaralingam S., Thirumalaiarasu V. (2010). Isolation and optimization of protease producing bacteria from halophilic soil. *Journal of Biosocial Science*.

[18] Lowry O. H., Rosebrough N. J., Farr A. L., Randall R. J. (1951). Protein measurement with the Folin phenol reagent. *The Journal of Biological Chemistry*.

[19] Imada A., Igarasi S., Nakahama K., Isono M. (1973). Asparaginase and glutaminase activities of micro-organisms. *Journal of General Microbiology*.

[20] Winkler U. K., Stuckmann M. (1979). Glycogen, hyaluronate, and some other polysaccharides greatly enhance the formation of exolipase by *Serratia marcescens*. *Journal of Bacteriology*.

[21] Rohban R., Amoozegar M. A., Ventosa A. (2009). Screening and isolation of halophilic bacteria producing extracellular hydrolyses from Howz Soltan Lake, Iran. *Journal of Industrial Microbiology and Biotechnology*.

[22] Jayachandra S. Y., Parameshwar A. B., Mohan R. K., Sulochana M. B. (2012). Characterization of extracellular hydrolytic enzymes producing extremely halophilic bacterium *Virgibacillus sp*. *World Journal of Science and Technology*.

[23] Coronado M.-J., Vargas C., Hofemeister J., Ventosa A., Nieto J. J. (2000). Production and biochemical characterization of an *α*-amylase from the moderate halophile *Halomonas meridiana*. *FEMS Microbiology Letters*.

[24] Li X., Yu H.-Y. (2012). Characterization of an organic solvent-tolerant *α*-amylase from a halophilic isolate, *Thalassobacillus* sp. LY18. *Folia Microbiologica*.

[25] Kim J., Dordick J. S. (1997). Unusual salt and solvent dependence of a protease from an extreme halophile. *Biotechnology and Bioengineering*.

[26] Giménez M. I., Studdert C. A., Sánchez J. J., De Castro R. E. (2000). Extracellular protease of *Natrialba magadii*: purification and biochemical characterization. *Extremophiles*.

[27] Studdert C. A., Herrera Seitz M. K., Plasencia Gil M. I., Sanchez J. J., de Castro R. E. (2001). Purification, biochemical characterization of the haloalkaliphilic archeon *Natronococcus occultus* extracellular serine protease. *Journal of General Microbiology*.

[28] Karbalaei-Heidari H. R., Amoozegar M. A., Hajighasemi M., Ziaee A.-A., Ventosa A. (2009). Production, optimization and purification of a novel extracellular protease from the moderately halophilic bacterium *Halobacillus karajensis*. *Journal of Industrial Microbiology & Biotechnology*.

[29] Dodia M. S., Joshi R. H., Patel R. K., Singh S. P. (2006). Characterization and stability of extracellular alkaline proteases from halophilic and alkaliphilic bacteria isolated from saline habitat of coastal Gujarat, India. *Brazilian Journal of Microbiology*.

[30] Ghasemi Y., Rasoul-Amini S., Kazemi A., Zarrini G., Morowvat M. H., Kargar M. (2011). Isolation and characterization of some moderately halophilic bacteria with lipase activity. *Microbiology*.

[31] Camacho R. M., Mateos J. C., González-Reynoso O., Prado L. A., Córdova J. (2009). Production and characterization of esterase and lipase from *Haloarcula marismortui*. *Journal of Industrial Microbiology and Biotechnology*.

[32] Camacho R. M., Mateos-Díaz J. C., Diaz-Montaño D. M., González-Reynoso O., Córdova J. (2010). Carboxyl ester hydrolases production and growth of a halophilic archaeon, *Halobacterium* sp. NRC-1. *Extremophiles*.

[33] Li X., Yu H. Y. (2012). Characterization of novel extracellular lipase from a halophilic isolate *Chromohalobacter* sp. LY7-8. *African Journal of Microbiology Research*.

[34] Kanlayakrit W., Boonpan A. K. (2007). Screening of halophilic lipase-producing bacteria and characterization of enzyme for fish sauce quality improvement. *Kasetsart Journal: Natural Science*.

[35] Wakayama M., Yamagata T., Kamemura A. (2005). Characterization of salt-tolerant glutaminase from *Stenotrophomonas maltophilia* NYW-81 and its application in Japanese soy sauce fermentation. *Journal of Industrial Microbiology and Biotechnology*.

[36] Koibuchi K., Nagasaki H., Yuasa A., Kataoka J., Kitamoto K. (2000). Molecular cloning and characterization of a gene encoding glutaminase from *Aspergillus oryzae*. *Applied Microbiology and Biotechnology*.

[37] Moriguchi M., Sakai K., Tateyama R., Furuta Y., Wakayama M. (1994). Isolation and characterization of salt-tolerant glutaminases from marine *Micrococcus luteus* K-3. *Journal of Fermentation and Bioengineering*.

